# Correlation Analysis of Genotypes and Phenotypes in Chinese Male Pediatric Patients With Congenital Hypogonadotropic Hypogonadism

**DOI:** 10.3389/fendo.2022.846801

**Published:** 2022-05-20

**Authors:** Yi Wang, Miao Qin, Lijun Fan, Chunxiu Gong

**Affiliations:** ^1^ Department of Endocrinology, Genetics and Metabolism, Beijing Children’s Hospital, Capital Medical University, Beijing, China; ^2^ Department of Endocrinology, Genetics and Metabolism, National Center for Children’s Health, Beijing, China

**Keywords:** congenital hypogonadotropic hypogonadism, clinical and genetic characteristics, oligogenicity, dual CHH, family history

## Abstract

Congenital hypogonadotropic hypogonadism (CHH) can be divided into Kallmann syndrome (KS) and normosmic HH (nHH). The clinical and genetic characteristics of CHH have been studied in adults, but less in pre-adults. The medical records of patients with CHH in our gonad disease database from 2008 to 2020 were evaluated. In total, 125 patients aged 0 to 18 years were enrolled in our study. KS patients had a higher incidence of micropenis compared with nHH (86.2% vs. 65.8%, p=0.009), and 7 patients (5.6%) had hypospadias. Among the 39 patients with traceable family history, delayed puberty, KS/nHH, and olfactory abnormalities accounted for 56.4%, 17.9%, and 15.4%, respectively. In total, 65 patients completed the hCG prolongation test after undergoing the standard hCG test, and the testosterone levels of 24 patients (22.9%) were still lower than 100 ng/dL. In 77 patients, 25 CHH-related genes were identified, including digenic and trigenic mutations in 23 and 3 patients, respectively. The proportion of oligogenic mutations was significantly higher than that in our previous study (27.7% vs. 9.8%). The most common pathogenic genes were *FGFR1*, *PROKR2*, *CHD7* and *ANOS1.* The incidence rate of the genes named above was 21.3%, 18.1%, 12.8% and 11.7%, respectively; all were higher than those in adults (<10%). Most mutations in CHH probands were private, except for W178S in *PROKR2*, V560I in *ANOS1*, H63D in *HS6ST1*, and P191L and S671L in *IL17RD*. By analyzing family history and genes, we found that both *PROKR2* and *KISS1R* may also be shared between constitutional delay of growth and puberty (CDGP) and CHH. L173R of *PROKR2* accounts for 40% of the CHH population in Europe and the United States; W178S of *PROKR2* accounts for 58.8% of Chinese CHH patients. Micropenis and cryptorchidism are important cues for CHH in children. They are more common in pediatric patients than in adult patients. It is not rare of Leydig cell dysfunction (dual CHH), neither of oligogenic mutations diagnosed CHH in children. Both *PROKR2* and *KISS1R* maybe the potential shared pathogenic genes of CDGP and CHH, and W178S in *PROKR2* may be a founder mutation in Chinese CHH patients.

## Introduction

Congenital hypogonadotropic hypogonadism (CHH, MIM 615267) is a common cause of absent puberty and adult infertility, with an incidence rate of 1 per 4000 new births (1). When associated with anosmia or hyposmia, it is also known as Kallmann syndrome (KS, MIM 147950). When associated with a normal sense of smell, it is termed normosmic CHH (nHH), which accounts for 50% of the cases (2). There are approximately 1200–1500 gonadotropin-releasing hormone (GnRH) neurons in the vertebrate hypothalamus that can synthesize and release GnRH. CHH is caused by a deficiency in the synthesis, release, or action of GnRH, resulting in insufficient secretion of gonadotropins, followed by gonadal dysfunction.

Congenital male hypogonadism is divided into three types according to clinical manifestations and hormone levels: central (hypothalamic-pituitary), primary (testicular), and combined forms (hypothalamic-pituitary and testicular, the so-called dual hypogonadism) (3, 4). As a disease paradigm of GnRH deficiency, CHH provides insight into the physiology and pathophysiology of the hypothalamic-pituitary-gonadal axis (HPG). A follow-up study of adult CHH patients treated with GnRH pumps reported that 11.1% (10/90) of the patients remained hypogonadotropic and hypogonadal. This suggests that the patients had both pituitary and testicular defects. Only 8.9% (8/90) of patients achieved spermatogenesis and normal T, but with hypergonadism, demonstrating poor testicular responsiveness to gonadotropins. Also, 5.6% (5/90) of patients had azoospermia but with adult testicular volumes and normal hormonal levels, indicating primary defects in spermatogenesis, which is referred to as dual CHH (5).

According to its pathophysiology, CHH is mainly divided into two types. During the fetal period, neurodevelopmental gene mutations cause disorders in the development, differentiation, or migration of GnRH neurons, usually causing KS. Defects in GnRH synthesis, release, or action in pituitary gonadotropin cells caused by neuroendocrine gene mutations usually lead to nHH (1). Many studies have found that CHH can be caused by gene defects that affect both neuronal development and the GnRH signaling pathway. Mutations in the same CHH-related pathogenic gene often cause phenotypic differences among patients or individuals in the same family; the low penetrance of most genes suggests that CHH is not a strictly monogenic disease (6, 7). Studies including large CHH cohorts have suggested that at least 20% of CHH cases are oligogenic (7, 8). However, our previous study involving 64 patients indicated that oligogenic mutations accounted for only 9.8% of the mutations (9).

Since the first KS-related pathogenic gene, *ANOS1* was cloned in 1991, many CHH-related pathogenic genes have been identified. In 2015, the European CHH consensus summarized 31 pathogenic genes, including X-chromosome-linked recessive, autosomal recessive, and dominant genes (1). At present, more than 90 candidate genes may be involved in the pathogenesis of CHH, and some newly reported genes have been confirmed in CHH patients. However, some genes involved in GnRH neuronal migration and axon formation in animal models have not been confirmed in CHH patients (8, 10–16). In our previous study, only 10 pathogenic genes were identified in patients with CHH (9).

Men with CHH exhibited decreased trabecular thickness and lower cortical bone area despite long-term hormonal treatment. Early treatment during adolescence may enhance trabecular outcomes, highlighting the importance of early diagnosis and interval (17). Therefore, this study aimed to evaluate the relationship between genotypes and phenotypes in pediatric CHH, thus providing more evidence for early diagnosis and intervention.

## Materials and Methods

### Ethical Considerations

The study was approved by the Ethics Committee of Beijing Children’s Hospital, Capital Medical University, and written informed consent was obtained from the parents or legal guardians of the patients. All necessary data involved in the study were available.

### Subjects

A total of 125 male patients of Chinese Han nationality aged 0–18 years, who were treated at the endocrine clinic of our hospital between 2008 and 2020, were enrolled. The patients were not related to each other.

The diagnosis was made based on clinical expression and laboratory investigations, including sex hormone levels (AMH and INHB), olfactory bulb magnetic resonance imaging (MRI), hCG test, chromosome karyotype, and genetic analysis. The testicular volume in all patients was evaluated using a Prader Willy orchydometer.

### Inclusion Criteria

The inclusion criteria were as follows: (1) No puberty initiation (testicular volume < 4 ml) with or without genitourinary malformation (micropenis, cryptorchidism and hypospadias), and baseline serum testosterone ≤ 20 ng/dl and gonadotrophins (LH, FSH) at a prepubertal levels, or with puberty stagnation. The LHRH stimulation test results could be used as a reference when bone age >12 years; (2) There are no occupying lesions of pituitary and hypothalamus on MRI; (3) KS or nHH is depended on questionnaire of anosmia/hyposmia, and MRI of olfactory bulb; (4) Follow-up was needed to rule out delay growth and puberty and isolated growth hormone deficiency (GHD); (5) Molecular genetic testing supported the diagnosis (All mutations were predicted to be pathogenic, likely pathogenic or uncertain).

### Exclusion Criteria

The exclusion criteria were as follows: (i) Any ascertained diseases (for example, chromosomal abnormality, trauma, surgeries, congenital adrenal hyperplasia (CAH), NR5A1-related disorders of sex development) or other ascertained diseases accompanied by sex agenesis (such as Prader-Willi syndrome (PWS), multiple pituitary hormone deficiency); (ii) Presence of chronic systemic diseases (for example, uraemia, thalassaemia, poorly controlled diabetes); (iii) Protein-energy malnutrition; (iv) Eating disorders (for example, anorexia nervosa, bulimia); (v) Intracranial lesions or pituitary tumors.

### The Diagnostic Criteria of Micropenis

Micropenis was diagnosed according to the criteria of the Chinese Journal of Pediatric Surgery in 2010 (18).

### Diagnostic Criteria of Dual CHH

After the hCG prolongation test, the testosterone (T) level was still less than 100 ng/dL and the patient was diagnosed with dual CHH. If the hCG prolongation test was not performed, the level of T was less than 100 ng/dL after treatment with GnRH (5–10 ug/90 min, 16 pulses/d) for half a year, and the patient was diagnosed with dual CHH. If the hCG prolongation test was not performed, GnRH treatment was provided for less than half a year, and the level of T was still greater than 100 ng/dL, testicular Leydig cells were considered to have a good response.

### hCG Standard and Prolongation Tests

HCG standard test and prolongation test were performed as previously described by Wang et al. (9).

### Hormone Detection

LH, FSH, and T levels were measured using an enzyme-enhanced chemiluminescence immunoassay (Immulite 2000; Siemens Corporation, Munich, Germany). Normal laboratory levels for T is 180-608ng/dl. INHB was measured using Chemiluminescence immunoassay (iflash 3000-c chemiluminescence immunoanalyzer, Shenzhen yahuilong Biotechnology Co., Ltd, Shenzhen, China), normal laboratory levels for INHB is 18.22-311.27pg/ml.

### DNA Sequence Analysis

A total of 51 patients underwent gonadal panel analysis, including 164 genes, and 44 patients underwent whole-exome sequencing. All 164 genes were screened by referencing the OMIM and HGMD databases. We input the keywords (“idiopathic GnRH deficiency”, “congenital hypogonadotropic hypogonadism”, “complex hypogonadism”, “Kallmann syndrome”, “gonad dysgenesis”, “micropenis”, “cryptorchidism”, “hypospadias” and “disorders of sex development”) in Pubmed. The genes included in targeted next-generation sequencing (NGS) are listed in **Supplementary Table 1**. DNA was extracted from the peripheral blood leukocytes of patients and their parents and/or siblings. A NEXTSEQ 500 sequencer (Illumina Corporation, San Diego, CA, USA) was then used to filter out all possible pathogenic missense, frameshift, and splice site mutations. Design primers and Sanger sequencing were used to verify mutations in the samples. Missense mutations were assessed according to the American College of Medical Genetics and Genomics (ACMG) rules (19), and both frameshift and splicing sites were considered pathogenic mutations.

For whole-exome sequencing (The work was performed in Beijing Mygenostics co., LTD, Beijing, China), process is as follows: 1)DNA Library Preparation: Approximately 2 mL peripheral blood (EDTA anticoagulant) of the patient was collected, and genomic DNA was extracted using QIAamp Blood Midi Kit (QIAGEN, Germany) according to the instructions. Paired-end sequencing libraries then were prepared using a DNA sampleprep reagent set 1 (NEBNext). Library preparation included end repair, adapter ligation and PCR enrichment, and was carried out as recommended by Illumina protocols; 2) Targeted genes enrichment and sequencing: The amplified DNA was captured use GenCap Whole-exome capture kit (MyGenostics GenCap Enrichment technologies). The capture experiment was conducted according to manufacturer’s protocol. The average sequencing depth > 100X, fraction of target covered with at least 10X > 95%. The enrichment libraries were sequenced on Illumina HiSeq X ten sequencer for paired read 150bp; 3)Bioinformatics analysis: After sequencing, the rawdata were saved as a FASTQ format, then followed the bioinformatics analysis: First, Illumina sequencing adapters and low quality reads (<80bp) were filtered by cutadapt. After quality control, the clean reads were mapped to the UCSC hg19 human reference genome using BWA (http://bio-bwa.sourceforge.net/). Duplicated reads were removed using picard tools and mapping reads were used for variation detection. Second, the variants of SNP and InDel were detected by GATK HaplotypeCaller, then using GATK VariantFiltration to filter variant. After above two steps, the data would be transformed to VCF format, variants were further annotated by ANNOVAR and associated with multiple databases, such as,1000 genome, ESP6500, dbSNP, EXAC, Inhouse (MyGenostics), HGMD, and predicted by SIFT, PolyPhen-2, MutationTaster, GERP++; 4)Variants Selected: In this course, five steps using to select the potential pathogenic mutations in downstream analysis: (i) Mutation reads should be more than 5, mutation ration should be no less than 30%; (ii) Removing the mutation, the frequency of which showed more than 5% in 1000g, ESP6500 and Inhouse database; (iii) If the mutations existed in InNormal database (MyGenostics), then dropped; (iv) Removing the synonymous. (v) After (i), (ii), (iii), if the mutations were synonymous and they were reported in HGMD, left them. When finished above jobs, the mutations which were left should be the pathogenic mutations. All mutations of minor allele frequency < 1% in East Asian people, and pathogenic, likely pathogenic, or uncertain mutations were possibly related to the disease.

### Statistical Analysis

Statistical analyses were performed using SPSS 26.0 (SPSS Inc., Chicago, IL, USA). When comparing the two groups, independent sample t-test is used for normal distribution, and Mann-Whitney U-test is used for non-normal distribution, Chi square test was used to compare the proportion of cryptorchidism between KS and nHH groups. It is considered to be statistically significance when p < 0.05.

## Results

### Clinical Characteristics

**Figure 1:**
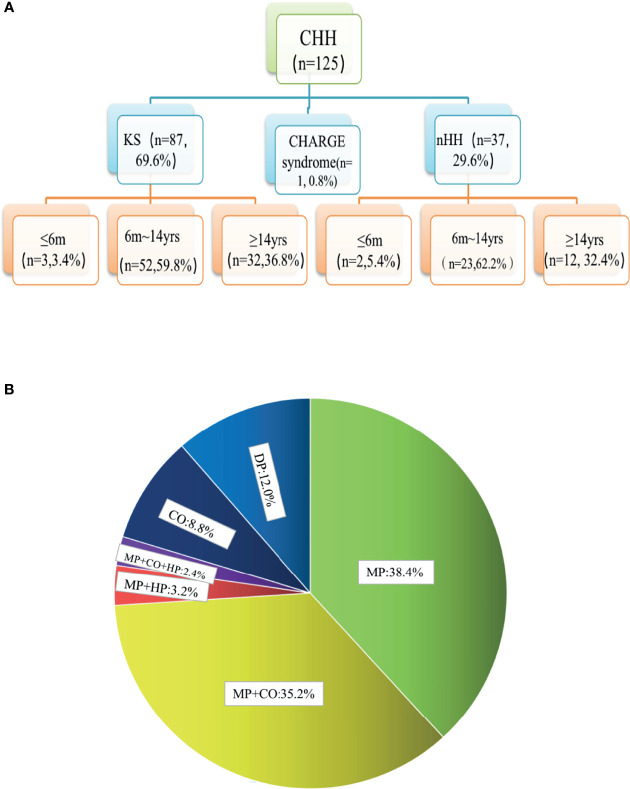
Age distribution and phenotypic proportion of 125 patients with CHH. **(A)** In our group of patients with KS and nHH, more than half were diagnosed before the age of 14, one third were diagnosed over the age of 14, and a few cases could be diagnosed within 6 months due to the lack of puberty. **(B)** 80.0% of the patients had micropenis, including those with micropenis (38.4%), micropenis with cryptorchidism (35.2%), micropenis with cryptorchidism and hypospadias (2.4%), micropenis with hypospadias (3.2%), 8.8% of the patients had cryptorchidism and 12.0% had absent puberty. MP, micropenis, CO, cryptorchidism; HP, hypospadias; DP, delayed puberty.

The chromosomes of all the patients were 46 XY and SRY (+). Combined with the phenotypes, physical signs, hormone levels, presence of puberty, olfactory bulb imaging, and genetic test results, a total of 125 cases of CHH were diagnosed, including 87 cases of KS (69.6%), 37 cases of nHH (29.6%), and 1 case of CHARGE syndrome. Only 5 patients (4%) were diagnosed 6 months after birth (**Figure 1A** and **Supplementary Table 2**). The results of 64 patients have been published elsewhere (9).

In total, 80.0% of the patients (100/125) had micropenis, including those with micropenis (38.4%, 48/125), micropenis with cryptorchidism (35.2%, 44/125), micropenis with cryptorchidism and hypospadias (2.4%, 3/125), micropenis with hypospadias (3.2%, 4/125), cryptorchidism (8.8%, 11/125), and absent puberty (12.0%, 15/125) (**Figure 1B** and **Table 1**).

**Table 1:** Genetic results of 7 patients with hypospadias.

No.	Date of birth	Diagnosis	Age* (yrs)	First gene	Nucleotide	Amino acid	Second gene	Nucleotide	Amino acid	Third gene	Nucleotide	Amino acid
1	2012/3/15	KS	0.42	HS6ST1	c.187C>G	p.H63D	SALL1	c.1984A>G	p.M662V			
2	2003/7/3	KS	3.42	ANOS1	c.958G>A	p.E320K						
3	2000/9/11	KS	9	FGF17	c.359C>T	p.P120L						
4	2005/8/30	KS	10.17	CHD7	c.3247A>G	p.T1083A	CHD7	c.6379G>A	p.A2127T	HS6ST1	c.1177G>A	p.D393N
5	2003/9/26	KS	12	ANOS1	c.1678G>A	p.V560I						
6	2017/5/6	nHH	3.25	PROKR2	c.472G>A	p.V158I	SPECC1L	c.694A>G	p.M232V			
7	2009/6/4	nHH	14	Negative								

*Age at diagnosis.

We further compared the clinical characteristics of KS and nHH and found that the incidence of micropenis in KS patients was higher. However, there were no significant differences in the incidence of cryptorchidism, testicular volume, length of micropenis, testosterone levels after hCG standard and prolongation tests (**Table 2**).

**Table 2:** Baseline clinical characteristics of 125 pediatric patients with CHH.

Items	KS (n = 87)	nHH (n = 38)	p value
Age at evaluation (yr)	13.0 (11.0, 14.8)	10.3 (3.9, 14.4)	0.08
Puberty (%)			
Partial	1.1	7.9	0.156
No	98.9	92.1	
Cryptorchidism (%)			
Unilateral	27.6	21.1	0.451*
Bilateral	21.8	21.1	
No	50.6	57.9	
Microphallus (%)			
Yes	86.2	65.8	0.009
Length of penis (cm)	3.1 (2.5, 4.0)	3.7 (2.5,4.9)	0.124
Testicular volume (ml)	1.5 (1.0, 2.0)	2.0 (1.0, 2.5)	0.534
T after hCG standard test (ng/dl)	44.2 (20.3, 115.2)	42.0 (20.0 156.5)	0.994
T after hCG prolonged test (ng/dl)	118.0 (64.7, 189.3)	138.4 (50.1, 185.3)	0.913

*Denotes the comparison of the proportion of cryptorchidism between KS and nHH groups. It is considered that there is a statistical difference When P < 0.05.

**Figure 2:**
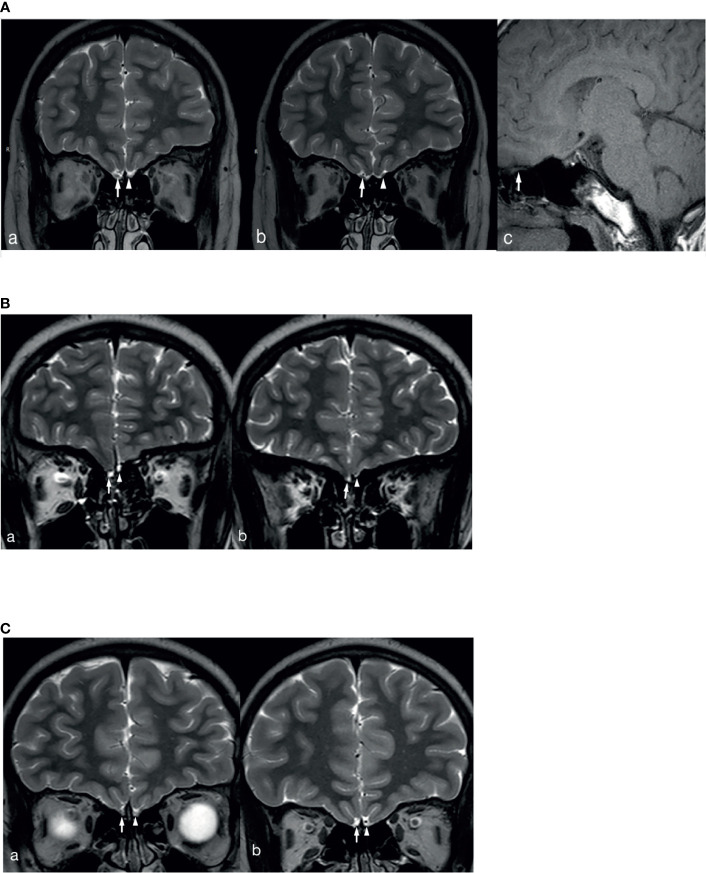
MRI images of 3 patients with KS. **(A)** The right olfactory bulb and olfactory tract of the patient are normally displayed (White long arrows in a and b show the right olfactory bulb and olfactory tract, white long arrow c shows sagittal position of right olfactory tract), the left olfactory bulb is not displayed (White short arrow in a), and the left olfactory tract (White short arrow in b) is smaller than the opposite side. **(B)** The bilateral olfactory bulbs and olfactory tracts of the patient are not clearly displayed (White long arrows in a and b show the right olfactory bulb and olfactory tract, white short arrows in a and b show the left olfactory bulb and olfactory tract), and the left olfactory sulcus is shallower than the opposite side (a and b). **(C)** The bilateral olfactory bulbs and olfactory tracts of the patient are not clearly displayed (White long arrows in a and b show the right olfactory bulb and olfactory tract, white short arrows in a and b show the left olfactory bulb and olfactory tract), and the bilateral olfactory sulcus is shallow (a and b).

In total, 2 patients with KS had left renal agenesis (one with *an ANOS1* mutation and the other without gene detection). In 1 patient with *an FGFR1* mutation, bimanual synkinesis was observed. A total of 65 patients with KS completed an MRI examination of the olfactory bulb, and 7.7% (5/65) reported hyposmia. However, no abnormal olfactory bulb, olfactory tract, or olfactory sulcus was found on the MRI. Based on the description and observation of olfaction by children and their parents, normal olfactory function was reported in 30.8% of the cases (20/65). Meanwhile, structural abnormalities of the olfactory bulb, olfactory bundle, and/or olfactory sulcus were observed on MRI, consistent with the results found in the literature and our previous reports. The MRI images of 3 patients with KS are shown in **Figure 2**. Patients could show structural abnormalities, for example, unilateral or bilateral olfactory bulb and olfactory tract cannot be displayed and shallow olfactory sulcus. However, on the other hand, some patients claim that they have normal smell, but the MRI examination is not perfected. These patients cannot be excluded the diagnosis of KS. Therefore, the proportion of KS in this study may be higher.

### Genetic Characteristics

**Figure 3:**
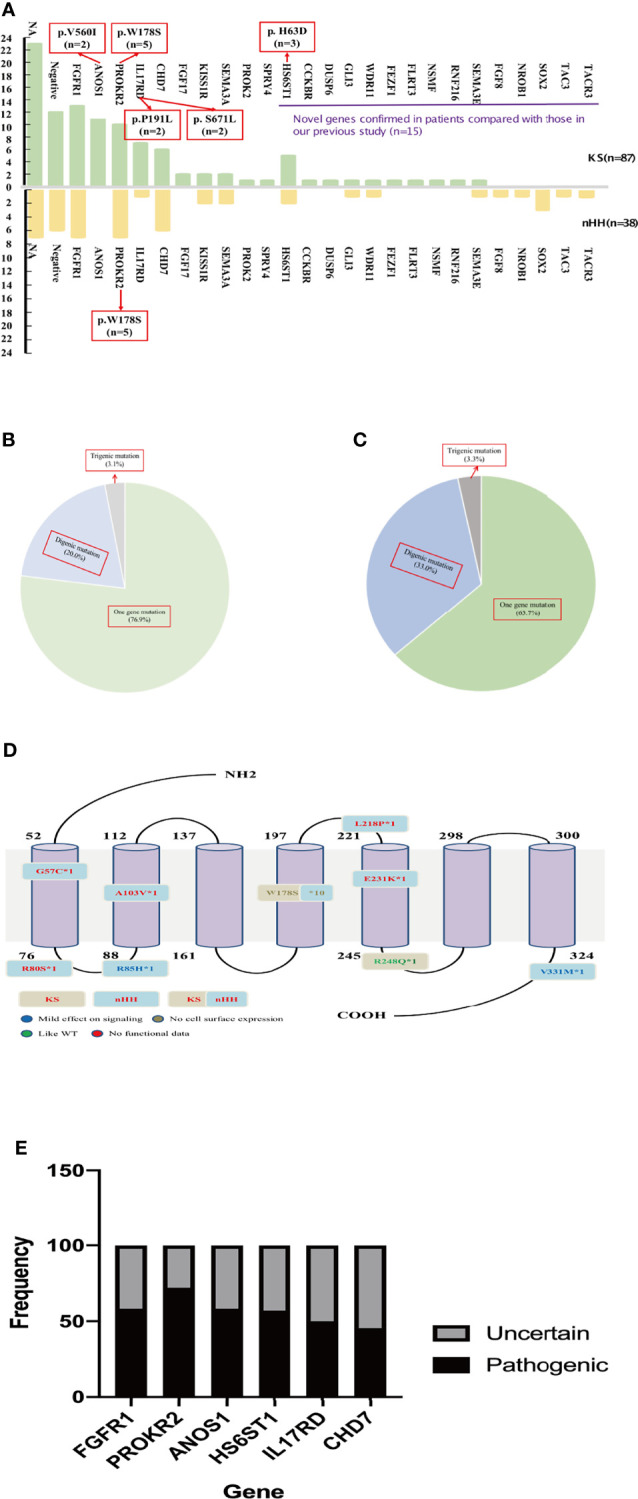
Gene analysis of 125 patients. **(A)** The gene distribution of patients with KS and nHH. As can be seen from the **Figure 2A**, 15 new genes were confirmed in patients compared with our previous report. Multiple genes (*ANOS1*, *PROKR2*, *IL17RD* and *HS6ST1*) were found to have repeated mutation sites. The gene of the patient with CHARGE syndrome was classified as nHH. **(B)** Digenic and trigenic gene mutations accounted for nearly a quarter of KS patients; **(C)** Digenic and trigenic gene mutations accounted for more than one third of nHH patients. **(D)** *PROKR2* was one of the most common genes in CHH patients, 18 mutation sites were found in 17 patients, mostly loctated in transmembrane region and intracellular segment, and W178S accounted for 58.8% (10/17 patients), which may could be as the founder mutation of Chinese CHH patients. **(E)** We further analyzed the ratio of common gene pathogenic/possible pathogenic mutations to uncertain mutation sites in this study, and found that 45.5% - 75.0% of the mutation sites were pathogenic or like pathogenic. For patients with uncertain mutation sites, we need longer follow-up to confirm its diagnosis and mutation pathogenicity.

The karyotype of all patients was 46; XY and the SRY gene was normal for NGS detection. A total of 94 patients underwent genetic testing: 25 CHH-related pathogenic genes were found in 81.9% of cases (77/94), 15 new CHH-related genes were confirmed in our patients compared to previous studies (**Figure 3A**), digenic mutations in 24.5% of cases (23/94), and trigenic mutations in 3.2% of cases (3/94) (**Table 3** and **Figures 3B, C**).

**Table 3:** Oligogenic mutations of 26 patients.

Case	Diagnosis	Number of gene	Number of Mutant Alleles	Gene	Nucleotide Change	Amino Acid Change	Mutation Type
1	KS	3	3	SEMA3E	c.760G>C	p.E254Q	missense
				CHD7	c.2824A>G	p.T942A	missense
				NSMF	c.188C>T	p.P63L	missense
2	KS	3	3	FGFR1	c.340-344delTTCTC	p.F114fs13	frameshift
				FEZF1	c.614C>T	p.A205V	missense
				FLRT3	m.2556G>A		noncoding region
3	nHH	3	4	PROKR2	c.533G>C	p.W178S	missense
				CHD7	c.*480_*481insAGGC		UTR
				CHD7	c.*480_*481insCAGTATGCT CGGGACGCCCTGGCTAAGAA CATCTACAGCCGCC		UTR
				FGF8	c.-72A>G		UTR
4	KS	2	3	FGFR1	c.801C>G	p.Y267Ter	missense
				PROKR2	c.743G>A	p.R248Q	missense
				PROKR2	c.533G>C	p.W178S	missense
5	KS	2	2	FGFR1	c.1034_c.1035del	p.S345Cfs54*	frameshift
				ANOS1	c.907G>A	p.V303I	missense
6	KS	2	2	FGFR1	c.736C>T	p.R246W	missense
				CHD7	c.8250T>G	p.F2750L	missense
7	KS	2	2	FGFR1	c.1704+1G>A		splicing site
				SPRY4	c.88C>T	p.R30W	missense
8	KS	2	2	PROKR2	c.533G>C	p.W178S	missense
				PROK2	c.301C>T	p.R101W	missense
9	KS	2	2	PROKR2	c.691G>A	p.E231K	missense
				IL17RD	c.192A>G	p.M658V	missense
10	KS	2	3	PROKR2	c.239G>A	p.R80H	missense
				PROKR2	c.169G>T	p.G57C	missense
				SEMA3A	c.1453-9delG		frameshift
11	KS	2	2	IL17RD	c.1319G>T	p.G440V	missense
				GLI3	c.1930G>A	p.G644R	missense
12	KS	2	3	IL17RD	c.572C>T	p.P191L	missense
				ANOS1	c.1654G>A	p.E552K	missense
				ANOS1	c.1062+1G>A	-	splicing site
13	KS	2	2	KISS1R	c.149C>A	p.A50E	missense
				CCKBR	c.1247G>A	p.R416H	missense
14	KS	2	3	CHD7	c.3247A>G	p.T1083A	missense
				CHD7	c.6379G>A	p.A2127T	missense
				HS6ST1	c.1177G>A	p.D393N	missense
15	KS	2	2	FGF17	c.580C>G	p.Q194E	missense
				CHD7	c.7912A>G	p.I2638V	missense
16	KS	2	2	FGFR1	c.1439T>G	p.L480X	truncation
				SEMA3A	c.1306G>A	p.V436I	missense
17	nHH	2	2	FGFR1	c.238C>T	p.R80C	missense
				SOX2	c.695C>A	p.T232N	missense
18	nHH	2	2	FGFR1	c.142G>A	p.G48S	missense
				GLI3	c.3286G>A	p.V1096M	missense
19	nHH	2	2	PROKR2	c.308C>T	p.A103V	missense
				SEMA3E	c.760G>C	p.E254Q	missense
20	nHH	2	2	PROKR2	c.533G>C	p.W178S	missense
				CHD7	c.6955C>T	p.R2319C	missense
21	nHH	2	2	SOX2	c.330C>A	p.Y110Ter	missense
				SEMA3A	c.1432G>A	p.E478K	missense
22	nHH	2	2	SOX2	c.6955C>A	p.T232N	missense
				CHD7	c.2656C>T	p.R886W	missense
23	nHH	2	2	HS6ST1	c.1177G>A	p.D393N	missense
				TAC3	c.107G>A	p.R36H	missense
24	nHH	2	2	KISS1R	c.929G>A	p.C310Y	missense
				NROB1	c.379G>A	p.A127T	missense
25	nHH	2	3	FGFR1	c.-55A>G		noncoding region
				FGFR1	c.1825-30G>A		intron
				PROKR2	c.533G>C	p.W178Se	missense
26	nHH	2	2	WDR11	c.386G>A	p.R129H	missense
				SEMA3A	c.2200C>T	p.R734W	missense

Only 5 patients carried digenic mutations in our previous study, while in this study, the proportion of digenic and trigenic mutations was significantly higher than that before (27.7% vs. 9.8%).*Means mutation leads to amino acid termination.

The most common mutations were *FGFR1* (20/94, 21.3%), *PROKR2* (17/94, 18.1%), *CHD7* (12/94, 12.8%), and *ANOS1* (11/94, 11.7%). Among the most common gene variants, there was no significant difference in the proportion of *FGFR1*, *PROKR2*, and *CHD7* variants between KS and nHH (all p>0.05).

Most mutations in CHH probands were private, except for several sites, such as W178S of *PROKR2* (n=5 in KS and nHH, respectively), V560I of *ANOS1* (n=2 in KS), H63D of *HS6ST1* (n=3 in KS), and P191L and S671L of *IL17RD* (n=2 in KS, respectively) (**Figure 3A**).

The oligogenicity of common autosomal dominant inherited pathogenic genes accounted for 50% (*FGFR1*, 10/20) and 33.3% (*CHD7*, 4/12). The oligogenicity of autosomal recessive inherited pathogenic genes accounted for 47.1% (*PROKR2*, 8/17, **Figure 3D**), and the oligogenicity of X-linked genes accounted for 9.1% (*ANOS1*, 1/11). Among the 12 patients with *CHD7* variants, only 1 patient (8.3%) was diagnosed with CHARGE syndrome. Among the 11 patients with KS caused by the *ANOS1* variant, 1 patient had exon 1 and 2 deletions, and another had all exon deletions.

We further analyzed the common pathogenic gene mutation sites according to ACMG. We found that 45.5%–75.0% of the mutation sites were pathogenic or likely pathogenic, except for 1 case of the *CHD7* variant being likely benign. The rest of these patients had either single-gene or oligogenic mutations (**Figure 3E**).

### Dual CHH and Genes

**Figure 4:**
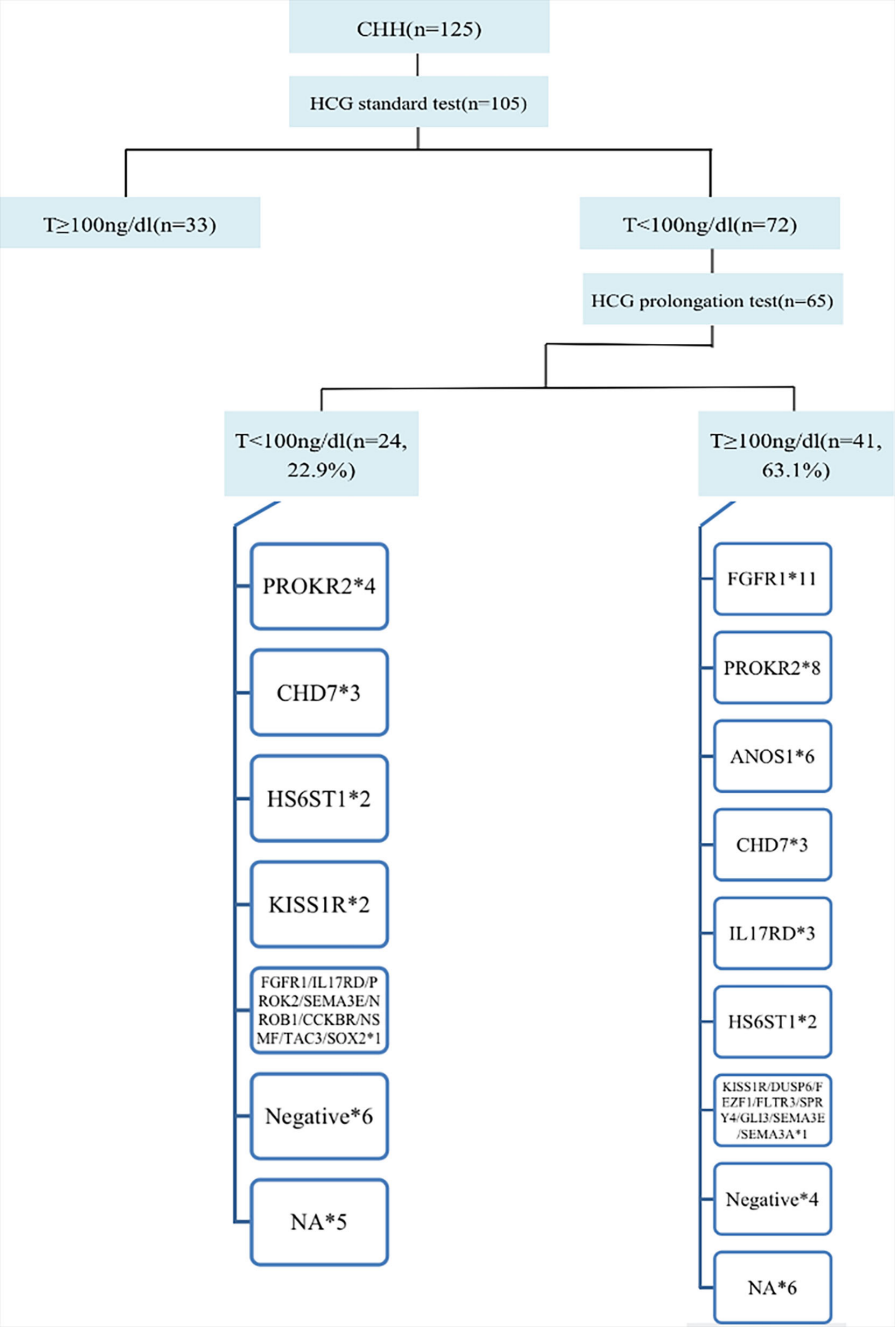
Diagnosis process and gene distribution of dual CHH. 65 patients continued to perform the hCG prolongation test, and T levels of 22.9% (24/105) patients were lower than 100ng/dl, suggesting that these patients also had testicular Leydig cell dysfunction (Dual CHH). In patients with dual and pure CHH, ratio of *FGFR1* mutation was 5.3% (1/19) vs. 31.4% (11/35), and there was no significant difference between the two groups (P = 0.138). *represents the number of cases with the same gene mutation.

In total, 105 of 125 patients completed the hCG standard test to evaluate testicular Leydig cell function. Of these 105 patients, 68.6% (72/105) had T<100 ng/dL, of whom 65 patients completed the hCG prolongation test, and 22.9% (24/105) of patients had T<100 ng/dL, suggesting testicular Leydig cell dysfunction. Therefore, at least 22.9% of the cases could be diagnosed as having dual CHH (**Figure 4**).

### Family History and Genes

**Figure 5:**
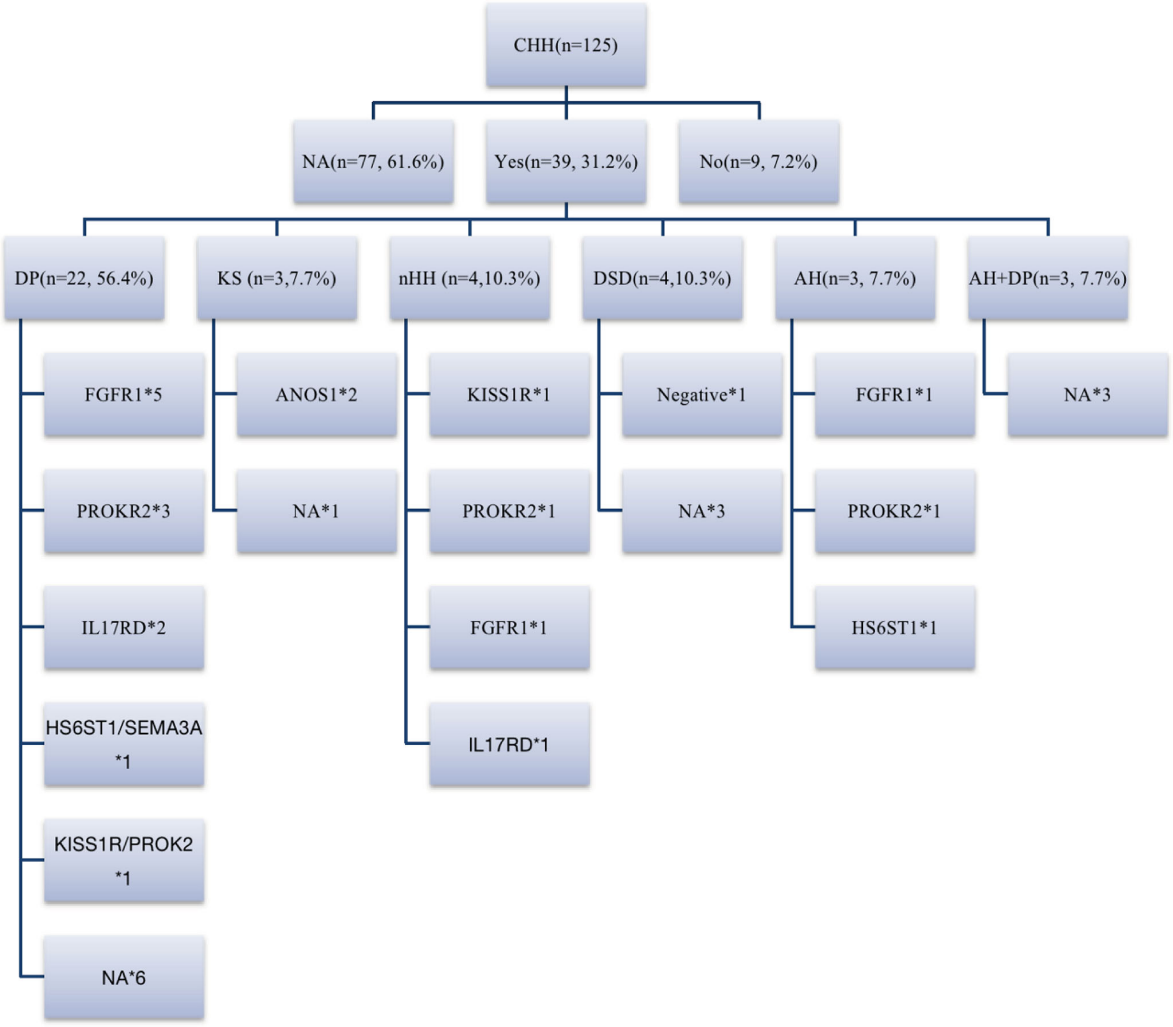
Family history and genes of patients with CHH. 31.2% (39/125) of patients could be traced back to positive family history, including DP, CHH, DSD, abnormal smell, abnormal smell combined with DP family history. 18% (7/39) of patients had KS/nHH family history, and more than half of patients had DP family history, *FGFR1*, *IL17RD*, *HS6ST1* and *SEMA3A* are shared genes of CHH and DP reported before, while in our cohort, we believe that *PROK2/PROKR2* and *KISS1R* are also potential shared genes. DP, delayed puberty; DSD, disorders of sex development; AH, anosmia or hyposmia. *represents the number of cases with the same gene mutation.

Among 39 patients with positive family histories, 56.4% (22/39) had a family history of delayed puberty (DP). In addition to the CHH-related genes (*FGFR1*, *HS6ST1*, *IL17RD*, and *SEMA3A*) reported in the literature for patients with DP (20, 21), 2 patients had *PROKR2* mutations in late-developing mothers (menarche at 16 years), and 1 carried *KISS1R* mutations in a late-developing father (first spermatorrhea at the age of 18–19 years), suggesting that *PROKR2* and *KISS1R* may also be shared genes of DP and CHH (**Figure 5**).

**Figure 6:**
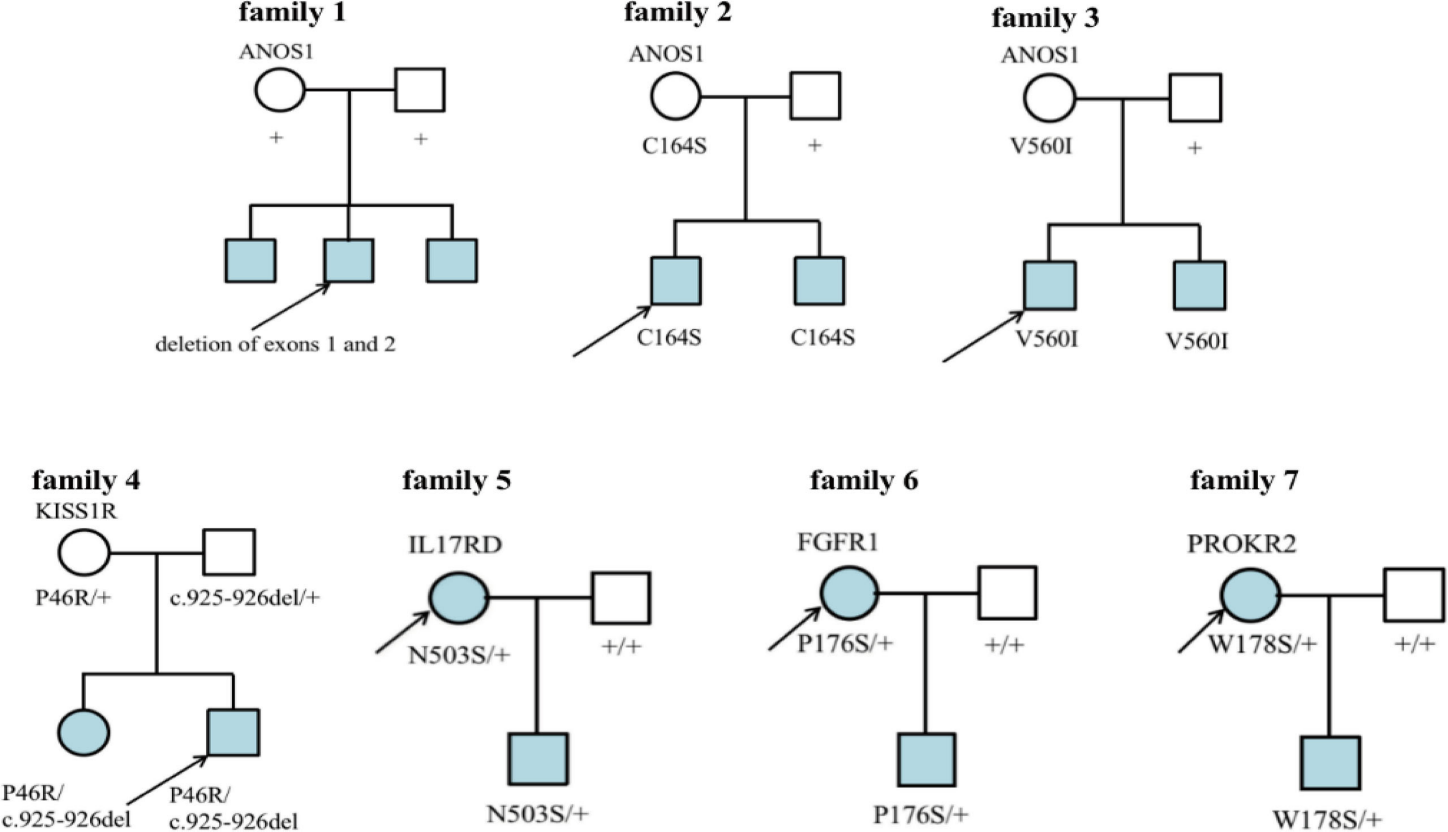
Family map of 7 CHH patients. Families 1-3 were KS patients, all probands harbored ANOS1 mutation. Families 4-7 were nHH patients. In families 5-7, mothers of the patiens were probands and gave birth to children after treatment, which further confirmed that N503S of *IL17RD*, P176S of *FGFR1* and W178S of *PROKR2* had relatively little effect on reproduction and could restore reproductive function after treatment. + denotes wild-type allele.

Seven families had CHH (**Figure 6**). In family 1, there were 3 KS brothers with *ANOS1* deletion of exons 1 and 2, and the eldest brother began to receive GnRH pump therapy in another adult hospital. In family 2, both KS brothers carried *ANOS1* missense mutation (C164S). In family 3, both KS brothers carried another *ANOS1* missense mutation (V560I). In family 4, there was 1 nHH patient with *K1SS1R* compound heterozygous mutations, and his elder sister carried the same mutation without puberty signs at the age of 17 years. In families 5-7, the three mothers were probands, and they were all treated with estrogen and progesterone to regulate their menstrual cycles. When they had fertility needs, they were administered GnRH pump therapy combined with assisted reproductive technology and carried three gene mutations (N503S of *IL17RD*, P176S of *FGFR1*, and W178S of *PROKR2)*.

## Discussion

This study summarized the clinical and genetic characteristics of 125 patients with CHH using CHH and DSD phenotypes (micropenis and cryptorchidism) and genetic testing as important cues for pediatric diagnosis of CHH. With an increase in the number of cases, more CHH candidate genes have been confirmed in patients. We found that digenic and trigenic variants accounted for 24.5% (23/94) and 3.2% (3/94) of the patients, respectively.

The most common mutant genes were similar to those reported in the literature, but mutations in each gene identified accounted for <10% of cases in previous studies (7, 8, 22–24), The proportion of common genes was significantly higher in our study: *FGFR1* (21.3%), *PROKR2* (18.1%), *CHD7* (12.8%), and *ANOS1* (11.7%). Among them, 10 cases (50.0%) of *FGFR1* and 7 cases (41.2%) of *PROKR2* mutations were oligogenic mutations. Previous studies have reported that *FGFR1* mutation may be related to hand and foot malformations in patients with CHH (22). However, in this study, only 1 patient with hand and foot malformations had *FGFR1* mutation; there was no obvious phenotypic-genotypic correlation, probably because more than half of the patients were oligogenic.

Previous studies on HH in adults reported that patients with *FGFR1* mutations had a high incidence of cryptorchidism, small testicular size, long treatment time for spermatogenesis, and low sperm concentration (25). However, in this study, compared with patients with non-*FGFR1* mutations, the incidence of cryptorchidism in patients with *FGFR1* mutations was not significantly higher, the testicular volume was not small, and the patients with *FGFR1* mutations were mainly pure CHH. However, further follow-up studies should be conducted because the diagnosis of pure CHH in this study was based on the T level after the hCG prolongation test, which may explain the difference.

INHB is a marker of the number of Sertoli cells and is usually lower than 30 pg/mL in male patients with complete CHH (1, 26). In some patients with partial CHH, the levels of INHB may overlap with those of DP and healthy controls (27, 28). In this study, the INHB test was performed in 65 patients, including all age groups, of which 45 (69.2%) had an INHB level of >30 pg/mL, suggesting that the function of testicular Sertoli cells in these patients was still good. Subsequent therapy with GnRH pump or hCG/HMG was more likely to promote spermatogenesis, consistent with the current spermatogenesis rate of 64.0%–80.3% in CHH patients after treatment (29, 30). Among the 39 patients with T>100 ng/dL after the hCG prolongation test, there were still 10 patients whose INHB level was <30 pg/mL, suggesting that some patients with a good response to Leydig cells may still have poor function of Sertoli cells. Therefore, evaluation of testicular cell function in patients with HH requires a multi-faceted and multi-index comprehensive evaluation.

However, in this study, 1 patient with three gene mutations (*SEMA3E/CHD7/NSMF*) was diagnosed with dual CHH. The patient was treated with a standard GnRH pump for 6 months, and the level of T was 112 ng/dL. After 12 months, it was observed that the patient had spermatorrhea and good sperm motility and concentration. Therefore, the percentage of patients diagnosed with dual CHH in this study with restored fertility after treatment requires further study. This phenomenon also suggests that there was a false-negative response to the short-term stimulation in the experiment.

In previous studies on adult cases, patients whose puberty was not induced by hormone therapy usually showed infertility when GnRH pump therapy was used to stimulate spermatogenic potential in adults. Therefore, for patients with CHH and their families, every gene mutation that causes GnRH deficiency should theoretically not be transmitted within the family. Recent studies have shown that a small number of gene mutation sites have strikingly high percentages; for example, the percentages of Q106R and R262Q in *GNRHR* were 44% and 29%, respectively (31–33) and W275X of *TACR3* was 36% (34–36). The L173R of *PROKR2* accounts for 40% of the CHH population in Europe and the United States but is rare in Asian populations (37–40). The recurrent mutation sites of several genes in this study were W178S of *PROKR2* (n=5 in KS and nHH), V560I of *ANOS1* (n=2 in KS), H63D of *HS6ST1* (n=3 in KS), and P191L and S671L of *IL17RD* (n=2 in KS). It was proven that *PROKR2* was one of the most common pathogenic genes in CHH, accounting for 17.9% (17/95) of genes in this study, W178S accounted for 58.8% (10/17). In another study of Chinese adult CHH patients, *PROKR2* mutations accounted for 13.3% (18/135), and W178S mutations accounted for 55.6% (10/18) (41). Combined with these two studies, W178S accounted for 57.1% of *PROKR2* mutations in the Chinese CHH population (20/35). Functional analysis showed that the mutant impeded receptor expression on the cell surface. W178S of *PROKR2* may be an ancient founder mutation, and it was not eliminated in the Chinese CHH population during evolution. The silencing of its effect on reproduction may be related to oligogenicity, or the mutant may revert during adulthood. However, in this study, the mother of 1 patient with the W178S mutation in *PROKR2* was a proband, suggesting that the mutation had a wide spectrum and that the patient could undergo germ cell maturation after treatment. Therefore, the complex mechanism of its effect on reproduction requires further study.

Of the 5 patients with single-gene mutations in *PROKR2* (W178S), 4 were diagnosed with dual CHH. Some patients harbor additional gene mutations. There was 1 patient with a repetitive mutation site *HS6ST1* (H63D), and another with *IL17RD* (N503S); they were also diagnosed with dual CHH, and *PROKR2* (W178S) and *IL17RD* (N503S) were carried by the proband’s mother simultaneously. However, the results of the hCG test showed that the Leydig cells were dysfunctional. It has been suggested that some patients with dual CHH diagnosed by the hCG test in this study may still recover their reproductive function after standard treatment. It could be that Leydig cells had not been stimulated by GnRH for a long time, which may have led to a slow response of the receptor. The curative effects in patients with these mutations and the relationship between the curative effect and gene mutations will be further investigated.

Hypospadias is caused by an abnormal urethral opening closure in the early stage of embryonic development, including the early hormone-independent stage (5–8 weeks) and hormone-dependent stage (8–12 weeks) (42). Before 12 weeks of gestation, the HPG axis has not been activated and cannot secrete gonadotropins, therefore, hypospadias and CHH may be two unrelated diseases. The Europe CHH consensus also believes that the existence of hypospadias could exclude the diagnosis of CHH.

In addition to our previous report, other HH patients with the hypospadias phenotype have also been reported (43–45). We analyzed 7 patients with hypospadias. In **Table 1**, 4 patients (patients 1-3 and 5) carried CHH-related pathogenic genes *HS6ST1*, *ANOS1* and *FGF17* respectively, and these three genes belong to “FGF8 synexpression group”. *HS6ST1* encodes a heparan sulfotransferase enzyme, which was required for anosmin-1 function *in vivo*, *ANOS1* could enhance *FGF* signaling by direct physical interactions with the FGFR-FGF-heparan sulfate proteoglycan complex on the cell surface. And *FGF17* has high homology to *FGF8*, while *FGF8* signaling is required for genital tubercle (GT) proximal-distal outgrowth, as abolishing *FGF8* or its receptors leads to GT agenesis in mice, resulting in hypospadias. Patient 4 harbored *CHD7* mutation, and *CHD7* plays an important role in gonad development and signaling, and mutation could cause hypospadias in previous study. Therefore, we believe that although the above genes are the pathogenic genes of CHH, they may also play an important role in the process of earlier penis development, and they may cause hypospadias after mutation, but the specific mechanism needs to be confirmed by further experiments. Two patients (patients 1 and 6) carried *SALL1* and *SPECC1L* mutations at the same time, which are involved in syndromes including hypospadias, but the role of these two mutations in the two patients may need to be further studied. On the other hand, endocrine disrupters like pesticide could also increase the risk of hypospadias (46), the phenomenon of CHH complicated with hypospadias needs more observation and research.

However, there are several limitations in the study. First of all, our olfactory judgment is based on the description and observation of olfaction by children and their parents, rather than olfactory test, some may be confusing. The disadvantage of the study is that only 94 patients (94/125, 75.2%) performed genetic testing, which may underestimate the rate of oligogenicity to CHH. On the other hand, due to the characteristics of CHH disease itself, CHH can only be confirmed after 18 yrs of age. Among our 125 patients, 27.2% are followed up to reach 18 years old, which requires longer follow-up to further verify the diagnosis of these patients.

## Conclusion

Micropenis, cryptorchidism, and molecular genetics are important factors for diagnosing CHH in pediatric patients. In this study, 15 new CHH genes were confirmed in patients. Oligogenic mutations accounted for 27.7% of all CHH patients, which may have been a mechanism of autosomal recessive hereditary genes or incomplete gene penetrance pathogenicity. KS due to *ANOS1* mutations is mostly caused by single genes, and *CHD7* mutations lead to isolated CHH. Approximately 25.0%–54.5% of common pathogenic gene mutations are uncertain, and their roles in the pathogenesis of CHH require further study. *PROKR2* and *KISS1R* may also share genes involved in DP and CHH. The *FGF* signaling pathway, represented by *FGFR1*, mainly causes CHH. The mothers of multiple probands carried the same mutation, and multiple gene mutation sites repeatedly appeared, suggesting that the effect of these mutation sites on reproduction was relatively slight. In this study, T after short-term hCG stimulation as an indicator of testicular function may be a false negative. We will further monitor the levels of T and INHB in patients with CHH after GnRH pump or hCG/HMG treatment and discuss dual CHH later.

## Data Availability Statement

The data presented in the study are deposited in the CNGB Sequence Archive (CNSA) of China National GeneBank DataBase (CNGBdb) repository, accession number CNP0002854.

## Ethics Statement

The studies involving human participants were reviewed and approved by the Ethics Committee of Beijing Children’s Hospital, Capital Medical University. Written informed consent to participate in this study was provided by the participants’ legal guardian/next of kin.

## Author Contributions

MQ and LF collected the data. YW analyzed the data and wrote the manuscript. CG revised the manuscript. All authors approved the final version of the manuscript.

## Funding

The study was supported by the Pediatric Medical Coordinated Development Center of Beijing Hospitals Authority (No. XTYB201808) and the Public Health Project for Residents in Beijing (Z151100003915103).

## Conflict of Interest

The authors declare that the research was conducted in the absence of any commercial or financial relationships that could be construed as a potential conflict of interest.

## Publisher’s Note

All claims expressed in this article are solely those of the authors and do not necessarily represent those of their affiliated organizations, or those of the publisher, the editors and the reviewers. Any product that may be evaluated in this article, or claim that may be made by its manufacturer, is not guaranteed or endorsed by the publisher.
